# Development of Ultrasound-Processed Poppy (*Papaver rhoeas* L.) Sherbet Enriched with Bee Bread Using Response Surface Methodology: Changes in Shelf Life

**DOI:** 10.1021/acsomega.4c03351

**Published:** 2024-06-18

**Authors:** Seydi Yıkmış, Nazan Tokatlı Demirok, Aksem Aksoy, Sema Sandıkçı Altunatmaz, Filiz Aksu, Rana Muhammad Aadil, Berna Erdal

**Affiliations:** † Department of Food Technology, Tekirdag Namık Kemal University, 59830 Tekirdag, Türkiye; ‡ Department of Nutrition and Dietetics, Faculty of Health Sciences, Tekirdag Namık Kemal University, 59030 Tekirdag, Türkiye; § Department of Food Engineering, Faculty of Engineering Architecture, Kafkas University, 36000 Kars, Türkiye; ∥ Food Technology Programme, Vocational School of Veterinary Medicine, Istanbul University-Cerrahpasa, 34320 Istanbul, Türkiye; ⊥ National Institute of Food Science and Technology, University of Agriculture, 38000 Faisalabad, Pakistan; # Department of Medical Microbiology, Faculty of Medicine, Tekirdag Namik Kemal University, 59030 Tekirdag, Türkiye

## Abstract

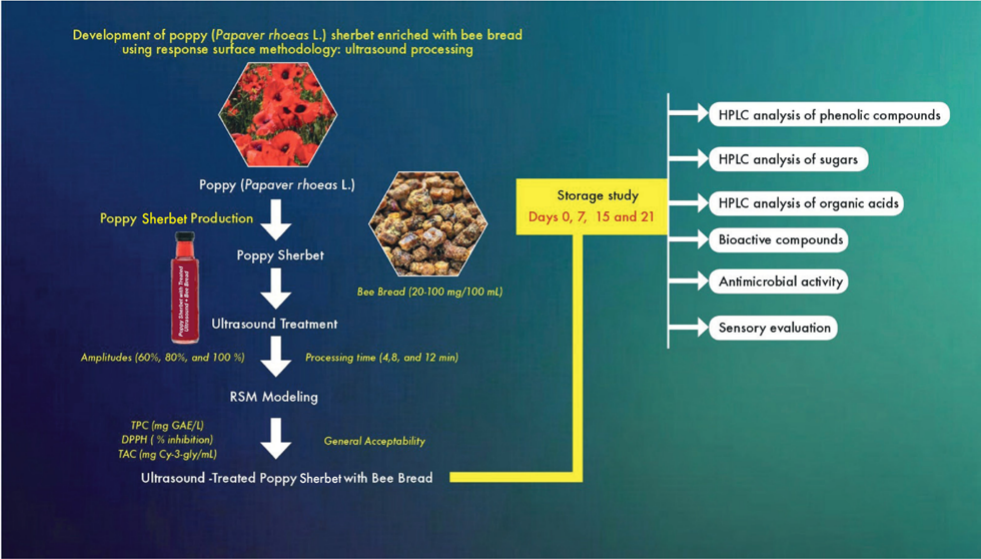

This research aimed to investigate the effects of ultrasound treatment on the quality characteristics of optimized functional bee bread-enriched poppy sherbet. Antioxidant activity capacity, antimicrobial activity, phenolic compounds, ascorbic acid, organic acid and sugar composition, and sensory properties were performed under storage conditions. The present research was the first to express the effect of ultrasound on the bioactive components in a functional poppy sherbet enriched with bread, using the response surface methodology (RSM) optimization. The maximum optimization, radical scavenging activity (DPPH), total phenolic content (TPC), total anthocyanin content (TAC), and general acceptability values were determined. When comparing the 0th- and 21st-day samples of bee bread-fortified functional poppy sherbets, it was observed that the TPC was decreased (*p* < 0.05). It was also noted that there was no significant decrease in the total flavonoids on day 21. In storage, a decrease in anthocyanin content was observed. Among phenolic compounds, gallic acid had the highest content. While citric acid was found in the highest amount of organic acid, sucrose (6.25 g/L) was found in the highest amount of sugar components 0th day, while MIC values against *Micrococcus luteus* were lower. The data from this study will be important input for future work.

## 1 Introduction

Poppy (*Papaver rhoeasL*.) is a red flowering and annual plants that usually grow in crop fields and meadows where red leaves are used to make poppy sherbet.^1^ Traditional drinks have been drunk for pleasure or to quench the thirst. They have also been used in medicine as healing agents.^2^ The poppy plant contains primarily phenolic and anthocyanins and some alkaloids such as coptisine, allotropine, berberine, and rhoeadine.^3^ As reported previously, poppies have antimutagenic, antigenotoxic, anticarcinogenic, anti-inflammatory, antitussive, soothing anxiety-related digestive problems, and antispasmodic properties.^4^

Bee products are natural foods/food supplements that people have been consuming for centuries.^5^ Bee bread, one of the most important functional products of the bees, is a fermented mixture (by lactic acid bacteria) of bee saliva, nectar, and plant pollen with a caramel color.^6,7^ Thanks to lactic acid fermentation, the bee bread is enriched with new nutrients and is more easily digestible.^8^ Bee bread has a rich macro- and micronutrients content such as protein, sugars, fatty acids, sterols, and fiber; phenolic acids, flavonoids, and phenylamides; vitamins (C, E, and K); and minerals (K, P, Ca, and Mg).^9^ Bee bread has been significantly studied by researchers and reported to have anticancer, antimicrobial, antioxidant, antiviral, and anti-inflammatory properties.^7^

Ultrasound is a nonthermal processing technology, which can be classified in the following ways: diagnostic ultrasound (1–500 MHz), high-frequency ultrasound (100 kHz–1 MHz), and power ultrasound (20–100 kHz).^10^ Ultrasound technology can shorten processing time, extend shelf life, and improve food quality^11^ There are a few studies on ultrasound-treated beverages in the literature review.^12−16^ Response surface methodology (RSM) is a collection of valuable statistical methods for process improvement, maintenance, and design. RSM determines the interaction between independent variables and dependent variables.^17,18^ The use of RSM in beverage optimization has been the focus of many recent studies.^19−22^

With the increasing consumer interest in natural, functional, and innovative beverages, there have been a few studies on poppy sherbet. However, this is the first study to optimize ultrasound-treated bee bread-fortified functional poppy sherbet. RSM was used to optimize the poppy sherbet enriched with bee bread. The aim is to study poppy sherbet enriched with bee bread properties using ultrasound technology. In addition, the objectives of the present study were as follows: (i) to optimize bee bread-fortified functional poppy sherbet and (ii) to determine antioxidant activity capacity (DPPH and CUPRAC), antimicrobial activity, phenolic compounds, organic acid and sugar composition, and sensory properties.

## 2 Materials and Methods

### 2.1 Preparation of Poppy Sherbet

Poppy leaves were purchased commercially Tekirdağ, Türkiye. The poppy sherbet was prepared at the Nutrition and Dietetics Laboratory of Tekirda Namık Kemal University, Tekirdağ, Türkiye. The formula used for the poppy sherbet was the same as that used in the study by Aydoğdu et al. In brief, the poppy sherbet was prepared with 0.15 g of lemon powder (citric acid), 4.29 g of sucrose sugar, and 0.26 g of poppy.^23^

### 2.2 Addition of Bee Bread and Ultrasound Treatment

Bee bread was purchased commercially, originating from the local market of Tekirdağ, Türkiye. The prepared sherbets were mixed with different amounts of bee bread (20–100 mg/100 mL). A 100 mL sample of poppy sherbet was then treated directly with ultrasound (26 kHz, Hielscher Ultrasonics model UP200 St, Germany). The ultrasound treatment parameters were time (4–12 min) and amplitude (60–100%). All samples were filtered after ultrasonic treatment and stored at 4 °C for further analysis. As a result of the optimization, the ultrasound-treated poppy seed sherbet with added bee bread (coded as UT-PS) was stored at +4 °C for 21 days, and the bee bread-enriched functional poppy sherbet samples were analyzed on the 0th, 7th, 14th, and 21st days of storage.

### 2.3 Modeling Procedure for RSM

RSM was analyzed using Minitab statistical analysis software (Minitab 18.1.1) to understand the effect of bioactive components and overall acceptability in preparing poppy sherbet with added bee bread. A three-level, three-factorial Box-Behnken design was chosen. The adequacy of the model was assessed based on the *R*^2^ and the adjusted *R*^2^ coefficients, the lack of fit tests, and the results of the analysis of variance (ANOVA). Bee bread (*X*_1_), ultrasound time (*X*_2_), and ultrasound amplitude (*X*_3_) were identified as independent variables. Dependent variables were chosen as the bioactive components and consumer acceptability. The study used three variable factors: the bee bread, ultrasound time, and ultrasound amplitude. Table 1 shows the levels and parameters of the variables in use: −1 is the minimum, 0 is the mean, and 1 is the maximum. A total of 15 experiments were carried out to optimize the process variables based on the coded values formulated in Table 1. To determine the relationship between the responses (bioactive ingredients, consumer acceptance) and the independent variables, the following polynomial was used (eq 1)
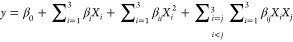
1The definition of this formula is as follows: *X_i_* and *X_j_* are independent variables, and the dependent variable (*y*) is the first-order (linear) equation coefficient (β*_i_*), the second-order equation coefficient (β*_ii_*), the two-factor cross-interaction coefficient (β*_ij_*), and the intercept term (β_o_).

**Table 1:** Experimental and Predicted Responses of RSM and Results of Poppy Sherbet^a^

				dependent variables							
independent variables				TPC (mg GAE/L)		DPPH (% inhibition)		TAC (mg Cy-3-gly/mL)		general acceptability	
no.	bee bread (mg/100 mL) (*X*_1_)	time (min) (*X*_2_)	amplitude (%) (*X*_3_)	experimental data	RSM predicted	experimental data	RSM predicted	experimental data	RSM predicted	experimental data	RSM predicted
1	20	4	80	57.91 ± 1.57	57.89	59.99 ± 1.19	59.99	42.45 ± 0.38	42.42	7.76 ± 0.71	7.75
2	100	4	80	58.85 ± 0.83	58.83	50.49 ± 0.71	50.49	33.16 ± 0.47	33.14	5.90 ± 0.25	5.89
3	20	12	80	53.29 ± 0.75	53.28	58.94 ± 0.83	58.95	38.68 ± 0.55	38.66	7.12 ± 0.30	7.11
4	100	12	80	60.65 ± 1.56	60.63	50.11 ± 1.05	50.12	34.11 ± 0.92	34.09	5.91 ± 0.25	5.90
5	20	8	60	56.24 ± 0.80	56.23	65.11 ± 0.92	65.12	43.19 ± 0.61	43.17	7.90 ± 0.34	7.90
6	100	8	60	59.53 ± 0.84	59.52	46.98 ± 0.66	46.98	30.37 ± 1.06	30.36	5.82 ± 0.25	5.81
7	20	8	100	49.78 ± 0.70	49.77	55.61 ± 1.50	55.62	36.97 ± 0.52	36.94	7.48 ± 0.32	7.48
8	100	8	100	54.80 ± 0.77	54.78	55.42 ± 0.78	55.42	35.94 ± 0.51	35.91	6.50 ± 0.28	6.49
9	60	4	60	61.29 ± 1.27	61.28	51.85 ± 1.44	51.85	34.50 ± 0.85	34.48	6.24 ± 0.26	6.23
10	60	12	60	62.07 ± 0.88	62.06	58.38 ± 0.83	58.39	37.84 ± 0.54	37.83	6.79 ± 0.29	6.79
11	60	4	100	57.87 ± 0.82	57.86	58.56 ± 0.83	58.57	38.92 ± 0.55	38.89	7.24 ± 0.31	7.23
12	60	12	100	54.29 ± 0.77	54.27	50.60 ± 0.72	50.61	32.77 ± 0.46	32.74	6.06 ± 0.26	6.05
13	60	8	80	60.14 ± 0.41	60.98	68.55 ± 0.97	68.90	42.58 ± 0.60	42.40	7.12 ± 0.30	7.29
14	60	8	80	61.28 ± 1.24	60.98	69.32 ± 1.20	68.90	41.34 ± 0.93	42.40	7.44 ± 0.16	7.29
15	60	8	80	61.56 ± 0.87	60.98	68.8 ± 0.97	68.90	43.34 ± 0.61	42.40	7.32 ± 0.31	7.29
UT-PS	43.43	7.39	73.33	60.41		69.35		43.52		7.58	
experimental values				57.22 ± 0.81		65.55 ± 0.93		40.16 ± 0.57		7.27 ± 0.25	
% difference				3.19		3.8		3.36		0.31	

a TPC: Total phenolic content; DDPH: radical scavenging activity; TAC: total anthocyanin content; GAE: gallic acid equivalent; Cy-3-gly: cyanidin 3-*O*-glucoside; UT-PS: Ultrasound-treated poppy sherbet.

### 2.4 Measurement of Bioactive Compounds and Antioxidant Activities

The calorimetric method determined the total flavonoid content (TFC).^24^ Results are expressed in milligrams of catechin equivalent (CE) per liter of TFC. Total phenolic content (TPC) was determined by the Folin–Ciocalteu method.^25^ Results are expressed in milligrams of gallic acid equivalent per liter (mg GAE/L) of bee bread-added poppy sherbet. The pH differential method was used to determine total monomeric anthocyanin (TMA).^26^ The absorbances were measured at 510 and 700 nm (λ_vis-max_). Results are expressed as milligrams of Cy-3-gly/mL. The antioxidant activity was evaluated using the 2,2-diphenyl-1-picrylhydrazyl (DPPH) radical. The method was previously described by Grajeda-Iglesias et al.^27^ A spectrophotometer (SP-UV/vis-300SRB, Melbourne, Australia) was used to measure the changes in absorbance at 517 nm. Trolox (0.0078–1 mg/mL) was used as a standard. Blank experiment was also performed without any sample to determine the absorbance of DPPH. Scavenging activity in this assay was expressed as IC_50_, which represents the sample concentration (μg/mL) required to inhibit 50% of free radical scavenging activity. Results were expressed in percent inhibition. The cupric reducing antioxidant capacity (CUPRAC) method was used to determine the total antioxidant activity of poppy sherbet with added bee bread by measuring its ability to reduce copper ions.^28^ The standard used was Trolox (0.0078–1 mg/mL). A blank experiment was also carried out without any sample to determine the absorbance of CUPRAC. CUPRAC in this assay was expressed as IC_50_. This sample concentration (μg/mL) is required to inhibit 50% of free radical scavenging activity. The results were expressed as % inhibition.

### 2.5 Determination of Phenolic Compounds Using HPLC-DAD

The analysis of phenolic compounds was performed on an Agilent 1260 Infinity chromatograph (Advanced Chromatography Technologies Ltd., Aberdeen, Scotland, C-18, ACE Generix column). The chromatograph was equipped with a diode array detector. The flow rate of the column was adjusted to 0.80 mL/min. The gradients were: 17% B at 0 min, 15% at 7 min, 20% at 20 min, 24% at 25 min, 30% at 28 min, 40% at 30 min, 50% at 32 min, 70% at 36 min, and 17% at 40 min. The gradient elution was performed by using solution A (phosphoric acid (0.1%) with water) and solution B (acetonitrile). The concentrations were expressed in μg/mL.^29^

### 2.6 Determination of Organic Acids and Sugars by HPLC-DAD

Organic acid and sugar content were analyzed using high-performance liquid chromatography (HPLC) with slight modifications to the method proposed by Coelho et al.^30^ The analysis was conducted with Agilent Technologies 1260 Infinity LC model (Santa Clara, CA). A 500 μL sample of poppy sherbet was filtered through a syringe filter (0.45 μm) and was injected (20 μL). The column used was Agilent Hi-Plex H (300 mm × 7.7 mm, 65 °C), and the RID flow cell was maintained at 35 °C. The applied flow rate was 0.6 mL/min, 20 min. The mobile phase consisted of H_2_SO_4_ (10.0 mM/L) in ultrapure water. Standards were injected. A diode array detector (DAD) was used for detection at 210 nm. Lactic acid, tartaric acid, malic acid, acetic acid, fumaric acid, oxalic acid, and succinic acids were determined. RID was used for the detection of the sugars fructose, turanose, maltose, glucose, sucrose, and xylose. The results are presented in grams per liter for sugars and organic acids.

### 2.7 Antibacterial Activity

#### 2.7.1 Bacterial Strains

For determining the antibacterial activity, Gram-negative bacteria: *Proteus vulgaris* (ATCC 3851), *Escherichia coli* (ATCC 2592), *Klebsiella pneumoniae* (ATCC 13883), and *Pseudomonas aeruginosa* (ATCC 27853) and Gram-positive bacteria: *Enterococcus faecalis* (ATCC 29212), *Micrococcus luteus* (ATCC 10240), *Bacillus cereus* (ATCC 11778), and *Staphylococcus aureus* (ATCC 25923) standard bacterial strains were used.

#### 2.7.2 Minimum Inhibitory Concentration (MIC) and Minimum Bactericidal Concentration

The MIC of the samples was determined using the CAMHB (Cation-adjusted Mueller–Hinton broth) medium and broth microdilution methods.^31^ Bee bread-fortified functional poppy sherbet samples were prepared with concentrations (100–0.19%). The bacteria were suspended in appropriate quantities in 0.5 McFarland (1.5 × 10^8^ CFU/ml) turbidity standard, diluted to 5 × 10^5^ CFU/mL, and added to a 96-well plate. After the cells were incubated at 37 °C for 18–20 h, a spectrophotometer was used to measure changes in absorbance at 630 nm. The MIC value was determined as the lowest concentration without growth.

#### 2.7.3 Disk Diffusion Test

The antibacterial activity of samples was determined using the Kirby Bauer disk diffusion method.^32^ Sheep blood agar (Catalogue Number: HM-09912; BES-LAB, Turkey) was used to inoculate bacteria strains. After incubation (37 °C for 16–18 h), Mueller Hinton agar was inoculated with a density-adjusted bacterial suspension (0.5 MacFarland). Disks (Bioanalyse BLK, CR) of 6 mm diameter were impregnated with 100 μL of samples and then placed onto the surface of inoculated plates (90 mm). After incubation (37 °C for 16–18 h), inhibition zone diameters (millimeters) were measured at the end of the incubation. Gentamicin (Oxoid, 10 μg) disks were used as a positive control.

### 2.8 Sensory Analysis

Following the optimization results, changes in the sensory properties of the poppy sherbet created after the application of the bee bread ultrasound amplitude and duration were investigated during the shelf life. Poppy sherbet enriched with bee bread (50 mL) was stored at 4 °C for 0, 7, 14, and 21 days. The overall acceptability of the sherbets was evaluated using 60 semitrained panelists (18 males and 42 females) at Tekirda Namık Kemal University, Türkiye. Random three-digit numbers were used to code all of the models. The sensory analysis environment was set up so that the panelists would not be physically influenced by each other. The analyses were conducted during daylight hours between 10:00 and 13:00. The panelists were given information on how to carry out the pretasting and testing procedures. Water was offered to the panelists to refresh their taste buds and to rinse their mouths after they had tasted the samples. The sensory attributes were rated on a nine-point hedonic scale (0–9).

### 2.9 Statistical Analysis

All analyses were carried out in triplicate. One-way ANOVA statistically examined the acquired data with SPSS 20.0 (SPSS Inc., Chicago). In the present study, Tukey tests were used to compare means using a significance level of *p* ≤ 0.05. The response surface methodology (RSM) utilized Minitab statistical analysis software (Minitab 18.1.1). The experiments were conducted with three replications.

## 3 Results and Discussion

### 3.1 Optimization of TPC, DPPH, TAC, and General Acceptability Parameters

Table 1 summarizes the data obtained from experimental and predictive results of poppy sherbet associated with dependent variables (TPC, DPPH, TAC, and general acceptability) and independent variables (bee bread (20 mg/100 mL–100 mg/100 mL, *X*_1_), time (4–12 min, X_2_) and amplitude (60–100%, X_3_)). A three-level, three-factor Box-Behnken Design (BBD) was applied to generate a total of 15 formulations (runs) to optimize the poppy sherbet.

**Figure 1:**
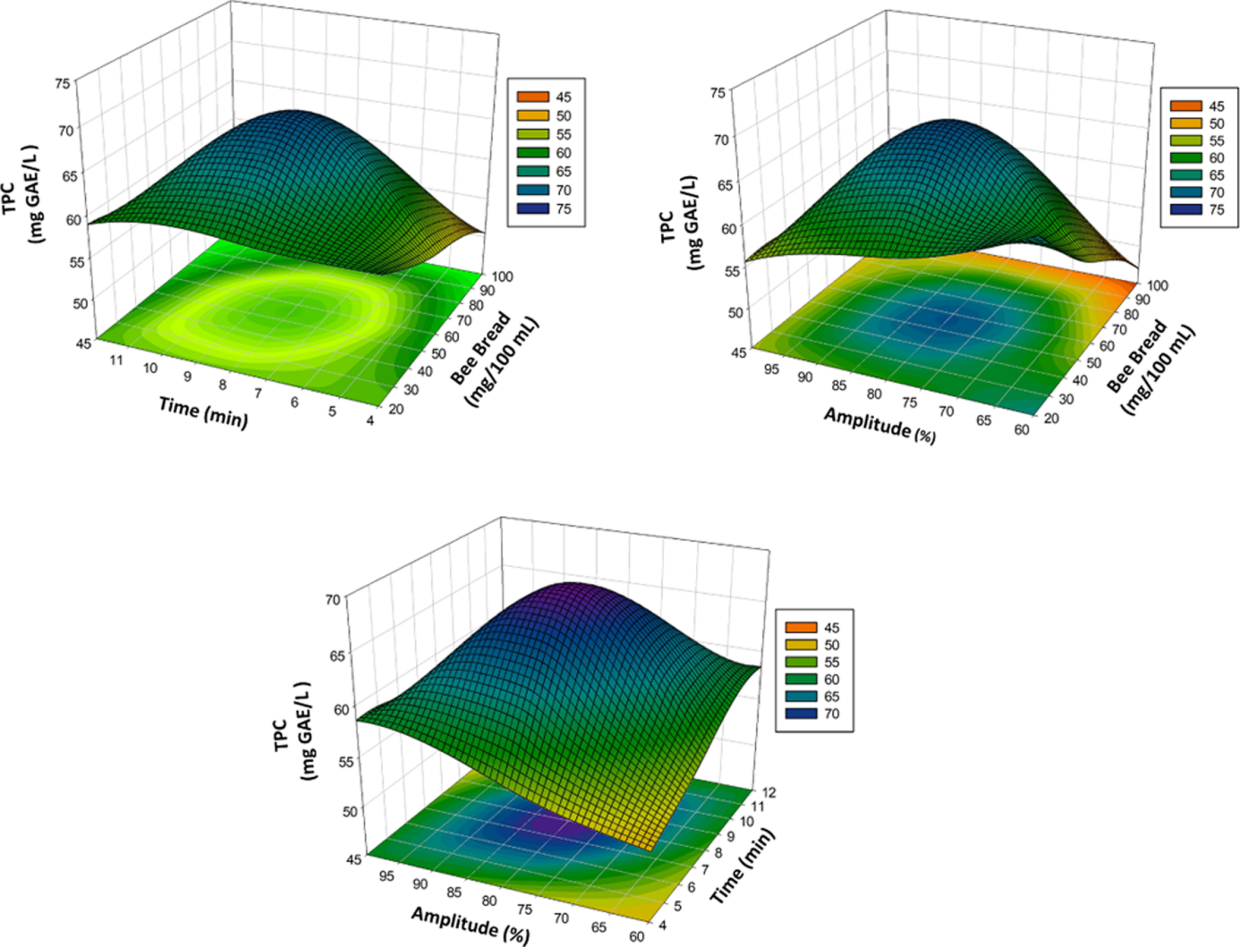
Three-dimensional response surface plots representing the effect of process variables on TPC (mg of GAE/L).

**Figure 2:**
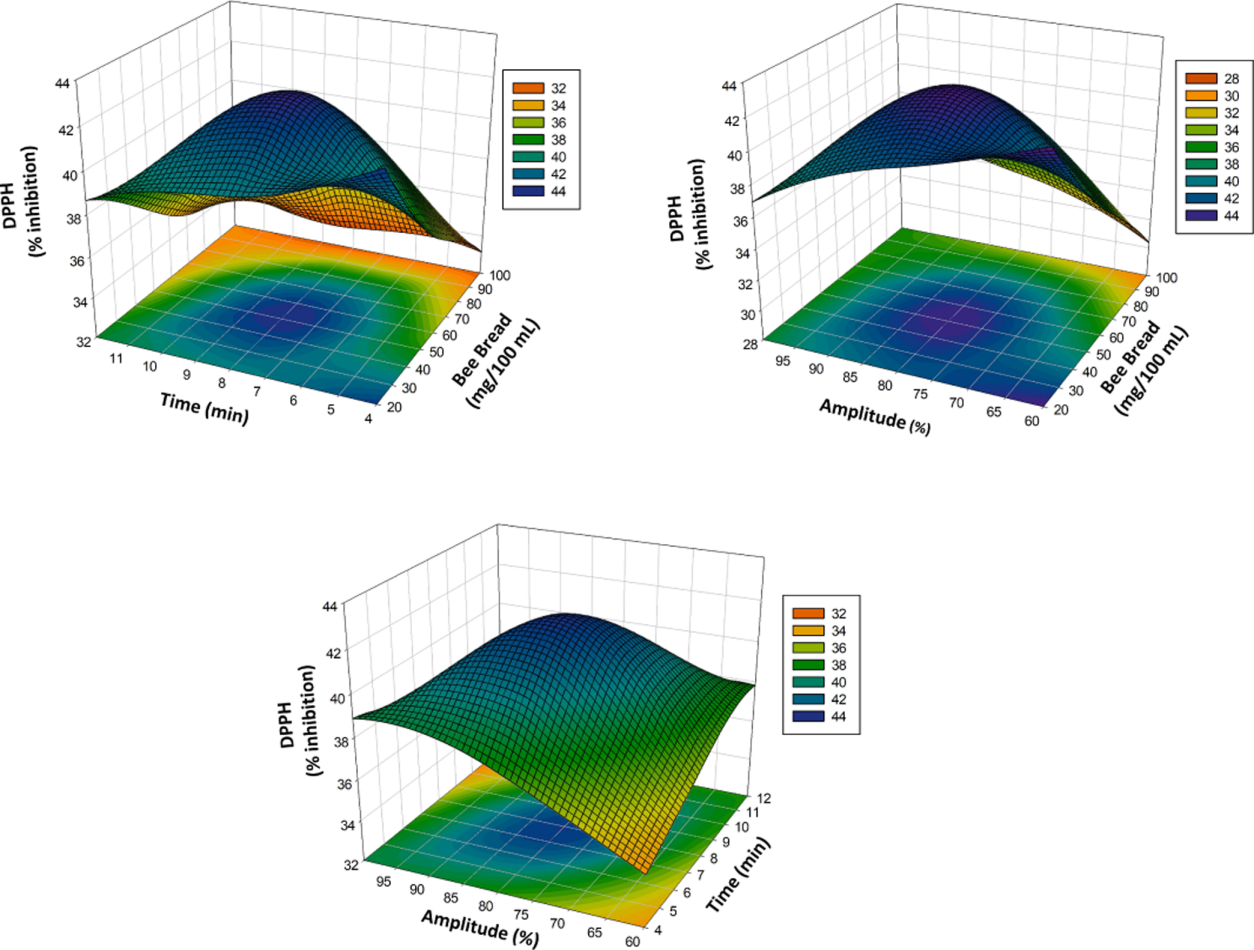
Effect of process variables on DPPH represented by 3D response surface plots.

**Figure 3:**
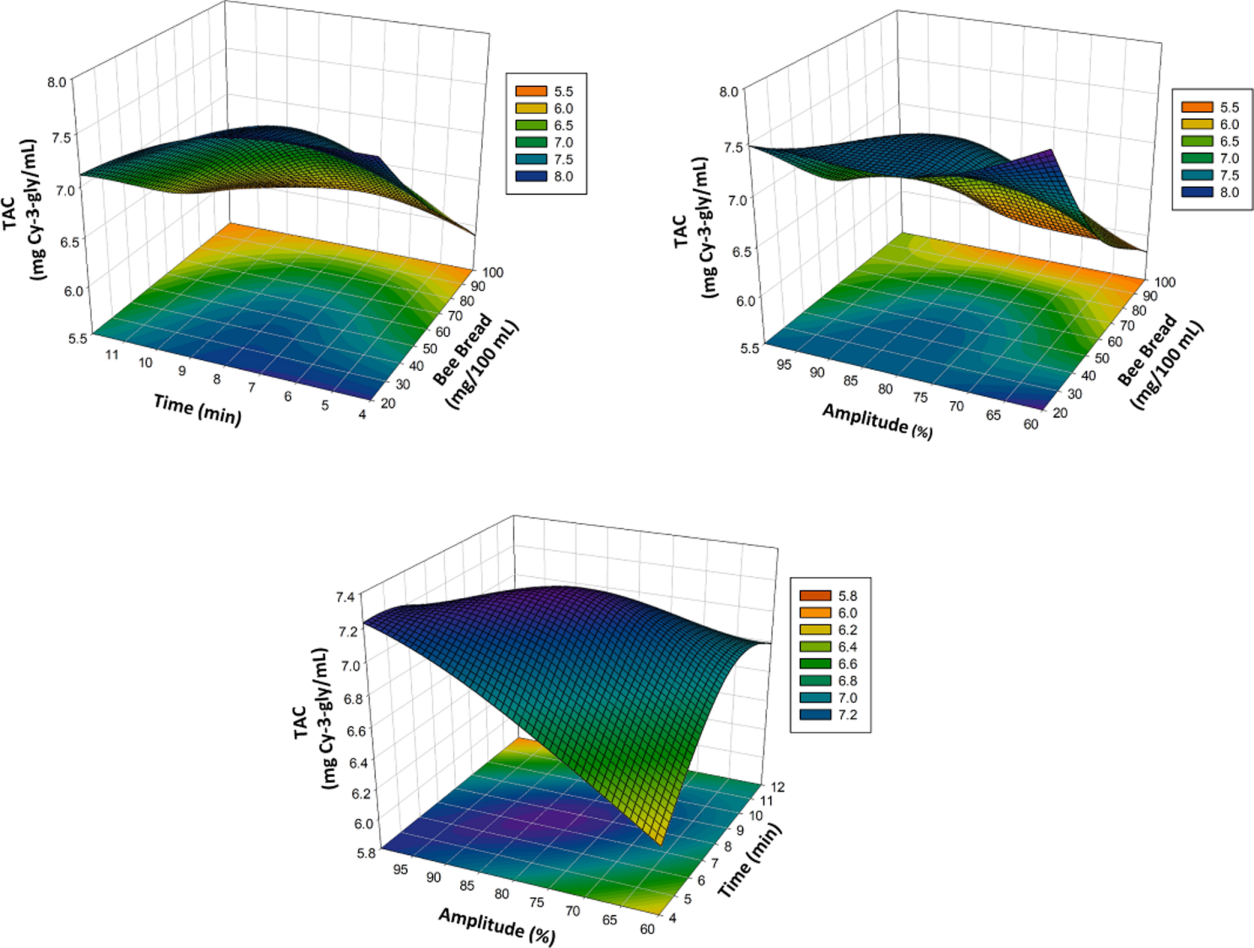
Effect of process variables on TAC (mg Cy-3-gly/mL) represented by 3D response surface plots.

**Figure 4:**
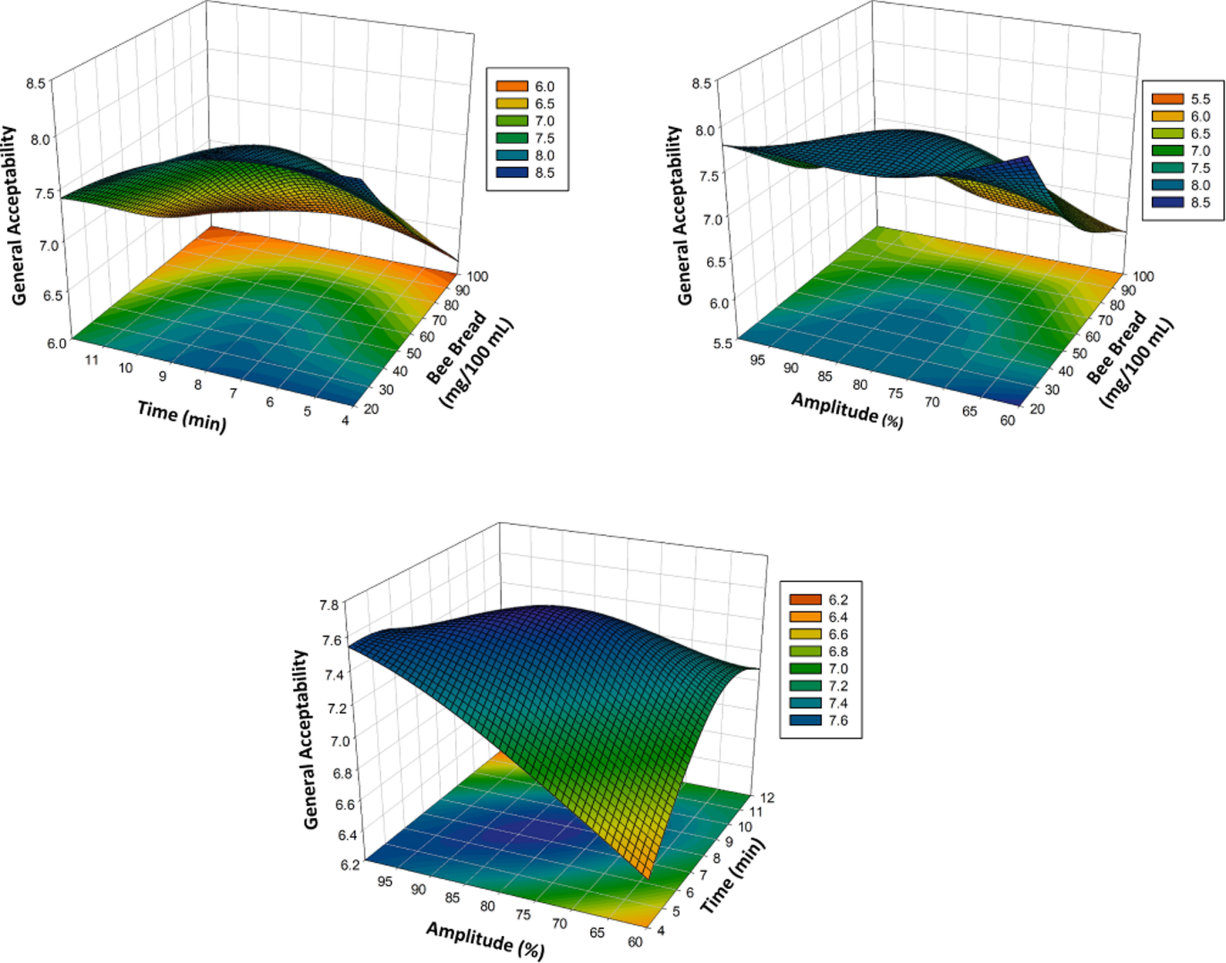
Three-dimensional response surface plots that show the effect of the process variables on the general acceptability

The analysis of variance (ANOVA) was carried out with the restriction that the *p*-value for the models was set at <0.05 (Table 2). The relation between bee bread (*X*_1_), time (*X*_2_), and amplitude (*X*_3_) for TPC, DPPH, TAC, and general acceptability are described in the following equation (eqs 2–5).
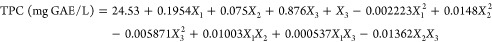
2
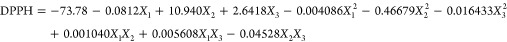
3
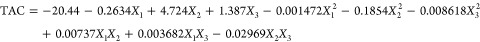
4
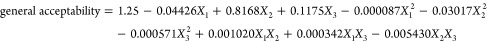
5The three-dimensional (3D) surface plots in Figures 1A–C, 2A–C, 3A–C, and 4A–C show the effects of bee bread, time, and amplitude on the TPC, DPPH, and TAC and general acceptability of the poppy sherbet. The TPC, DPPH, TAC, and general acceptability significantly affected the *X*_1_, *X*_2_ linear terms, that is, bee bread content and ultrasound treatment time. Some two-way interaquadraticuterms (*X*_2_*X*_3_) and some quadraiterm as *X*_1_^2^ and *X*_3_^2^ also showed a significant effect (*p* < 0.05) on TPC, DPPH, TAC, and general acceptability. Table 1, which shows the highest TPC value (62.07 ± 0.88 mg GAE/L) with the treatment of bee bread content 60 mg/100 mL, ultrasound time 12 min, and ultrasound power 60 amplitude (Run No. 15), as well as shows the lowest TPC value (49.78 ± 0.70 mg GAE/L) with the treatment of bee bread content 20 mg/100 mL, ultrasound time 8 min, and ultrasound power 100 amplitude (Run No. 7). The highest TAC value (43.19 ± 0.61 mg Cy-3-gly/mL) was obtained with the treatment of bee bread content 20 mg/100 mL, ultrasound time 8 min, and ultrasound power 60 amplitude (Run No. 5). The TAC was reduced when the bee bread content was increased. Habryka et al. indicated that taste acceptability decreased with increasing addition of bee bread. This is similar to the results of the present study.^33^Table 1 shows the highest general acceptability value (7.90 ± 0.34) with the treatment of bee bread content 20 mg/100 mL, ultrasound time (8 min), and ultrasound power (60 amplitude) (Run No. 15). The model was shown to be highly consistent by the F-value (75.790) and the low p-value (*p* < 0.05) for the general acceptance parameter. Aydoğdu et al. found TPC (47.36 ± 1.01 mg GAE/100 mL) and DPPH values (56.06 ± 0.45%) of optimized poppy sherbet using citric acid, sucrose, and dried poppy flower.^23^ Specifically, increasing time and amplitude had a negative effect on TPC, consistent with a previous study by Cheng et al. that showed decreasing TPC in ultrasound-treated mandarin (*Citrus unshiu*) juice.^34^ Different results obtained from the studies may be related to the ultrasound process, ultrasound power amplitude, ultrasound processing time, and type, volume, quantity, and matrix of food.

**Table 2:** Analysis of Variance of Independent Values Obtained from the RSM^a^

		TPC (mg GAE/L)		DPPH (% inhibition)		TAC (mg Cy-3-gly/mL)		general acceptability	
source	DF	*F*-value	*P*-value	*F*-value	*P*-value	*F*-value	*P*-value	*F*-value	*P*-value
model	9	88.980	0.000	1362.200	0.000	67.220	0.000	75.790	0.000
linear	3	148.970	0.000	915.770	0.000	81.620	0.000	157.650	0.000
*X*_1_	1	152.430	0.000	2721.760	0.000	234.650	0.000	450.940	0.000
*X*_2_	1	17.440	0.009	16.410	0.010	9.700	0.026	18.730	0.008
*X*_3_	1	277.050	0.000	9.140	0.029	0.520	0.503	3.280	0.130
square	3	94.720	0.000	2451.950	0.000	68.810	0.000	32.700	0.001
*X*_1_^2^	1	206.340	0.000	2557.280	0.000	50.070	0.001	6.890	0.047
*X*_2_^2^	1	0.920	0.381	3337.030	0.000	79.460	0.000	82.300	0.000
*X*_3_^2^	1	89.980	0.000	2584.890	0.000	107.310	0.000	18.420	0.008
2-way interaction	3	23.250	0.002	718.890	0.000	51.230	0.000	37.030	0.001
*X*_1_* *X*_2_	1	45.510	0.001	1.790	0.238	13.590	0.014	10.190	0.024
*X*_1_* *X*_3_	1	3.270	0.131	1304.460	0.000	84.880	0.000	28.680	0.003
*X*_2_* *X*_3_	1	20.980	0.006	850.410	0.000	55.210	0.001	72.200	0.000
error	5								
lack-of-fit	3	0.000	1.000	0.000	1.000	0.000	1.000	0.000	1.000
pure error	2								
total	14								
*R*^2^		99,38%		99.96%		99.18%		99.27%	
adj *R*^2^		98,26%		97.89%		99.70%		97.96%	
pred. *R*^2^		98,60%		99.91%		98.16%		98.36%	

a *X*_1_: Bee Bread; *X*_2_: Time; *X*_3_: Amplitude; DF: degrees of freedom; TPC: total phenolic content; DDPH: radical scavenging activity; TAC: total anthocyanin content; GAE: gallic acid equivalent; Cy-3-gly: cyanidin 3-*O*-glucoside.

The maximum values for optimization, DPPH, TPC, TAC, and general acceptability were 60.41 mg GAE/100 mL, 69.35%, 43.52 mg Cy-3-gly/mL, 7.58 at 7.39 min, and 73.33 amplitude at 43.43 mg/100 mL bee bread content, respectively. Comparison between the RSM and repeated experimental values of UT-PS gave 96.72, 96.72, 92.28, and 95.91% for TPC, DPPH, TAC, and total acceptability, respectively.

### 3.2 Bioactive Compounds

**Figure 5:**
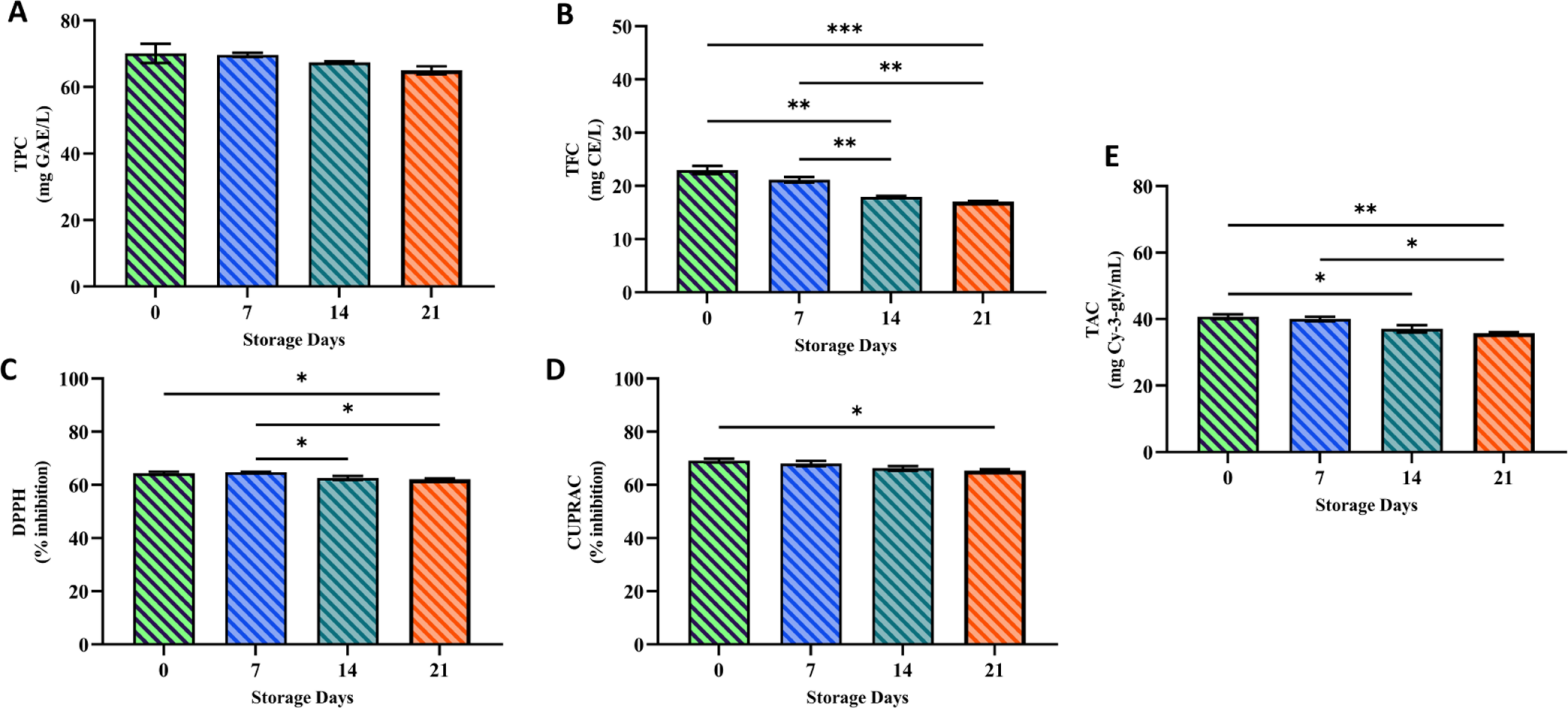
Total phenols (A), total flavonoids (B), radical scavenging activity (C), cupric reductant antioxidant capacity (D), and total monomeric anthocyanins (E) of the samples. The letters at the top of the bars indicate statistically significant differences. Two replicates were used for analyses (*p < 0.05; **p < 0.01; ***p < 0.001).

Phenolics are the most common secondary metabolic products in plants with various biological activities. It has antioxidant, anti-inflammatory, antibacterial, and antitumor activities.^35,36^ The results of TPC and TFC values on days 0, 7, 14, and 21 in the bee-butter-fortified functional poppy sherbets are given in Figure 5. Considering the total phenolic content, it was determined that it was 70.12 ± 2.91, 69.67 ± 0.63, 67.38 ± 0.37, and 65.04 ± 1.22 mg GAE/L on days 0, 7, 14, and 21, respectively. When comparing the 0th- and 21st-day samples of bee bread-fortified functional poppy sherbets, a reduction in the total phenolic content was observed (*p* < 0.05). In this research, a high correlation was found (*r* = 0.94) between total phenolic content and antioxidant activity of poppy sherbet. Similarly, several other studies have shown a positive high correlation between total phenolic content and antioxidant activity.^37,38^ Pearson’s positive coefficient of determination (*R*^2^) for predicting the TPC was significantly correlated with TAC (0.98). This finding differs from Habryka et al. who found that increasing bee bread content improves TAC value in honey. They also indicated that as the addition of bee bread increases, the acceptability of the taste decreases, which is similar to the findings of the present study.^33^ Marsoul et al. indicated that the TPC of *P. rhoeasL*. extracts were 95.4 ± 2.42 and 165.4 ± 3.84 mg GAE/g. Compared to our study, these values are quite high.^4^ Aydoğdu et al. similarly detected a decrease in phenolic contents of optimized poppy sherbet samples during storage (0, 10, 20, and 30 days were 47.36 ± 1.01, 46.87 ± 0.48, 43.76 ± 0.53, and 39.66 ± 0.67 mg/L, respectively).^23^ It is thought that this difference arises from the addition of bee bread and the method used in sherbet production. It is stated that the concentration method and storage temperature significantly affect the bioactivity of the sherbet.^39^ Flavonoids are the largest group of phenolic compounds found in plants.^40,41^ These compounds are considered to be very important components in various nutraceutical, pharmaceutical, medical, and cosmetic applications due to their various effects such as antioxidant, antiviral, antibacterial, anti-inflammatory, and antiallergic potentials.^42^ TFC in the sample during the storage period were determined as 22.99 ± 0.78, 21.16 ± 0.53, 17.93 ± 0.19, and 17.05 ± 0.14 mg CE/L, respectively. In the 14th-day sample, TFC decreased significantly. However, it was noted that there was no significant decrease in the total amount of flavonoids on the 21st day. A positive correlation (*r* = 0.92) was detected between total flavonoids and DPPH. It was determined that there was a high positive correlation (*r* = 1) between total flavonoids and CUPRAC. Similarly, Aydoğdu et al. reported that their TFC values were 16.69 ± 0.40, 15.54 ± 0.37, 13.01 ± 0.30, and 12.28 ± 0.21, respectively.^23^ This difference may be due to the different methods of adding bee bread and making the sorbet. However, consistent with the results of the above-mentioned study, a partial decrease in the total phenolic content during storage was observed in our study. When our study results were evaluated overall, it was observed that there was a partial decrease in the TPC and TFC values during the storage period.

### 3.3 Antioxidant Activity during Storage

Poppy has antiradical activity because it contains bioactive compounds (TFC and TPC).^4^ Studies have emphasized that bee bread also has antioxidant potential.^43,44^ The results of the DPPH and CUPRAC values on days 0, 7, 14, and 21 in the bee bread-fortified functional poppy sherbet samples are given in Figure 5. The percentage of DPPH inhibition in the sample during the storage period was determined as 64.39 ± 0.59, 64.79 ± 0.19, 62.62 ± 0.74, and 62.12 ± 0.3%, respectively. It was found that the DPPH and CUPRAC inhibition percentages were statistically higher for the sample stored on day 0 than the sample stored on day 21 (*p* < 0.05). DPPH and CUPRAC have a high positive correlation (*r* = 0.92). Isbilir and Sagiroglu found that the scavenging effects of *P. rhoeasL*. leaves extracts (water and ethanol) at a concentration of 800 μg/mL against DPPH radical were 88.46 ± 0.08 and 86.81 ± 0.37%, respectively, and they suggested that *P. rhoea* L. leaves have the potential for use as a natural antioxidant.^45^ In another study examining the TFC and TPC of different extracts of *P. rhoeasL*., extracts exhibited a strong scavenging activity against DPPH radicals, and this rate was over 80%.^46^

### 3.4 Total Monomeric Anthocyanin Content during Storage

**Figure 6:**
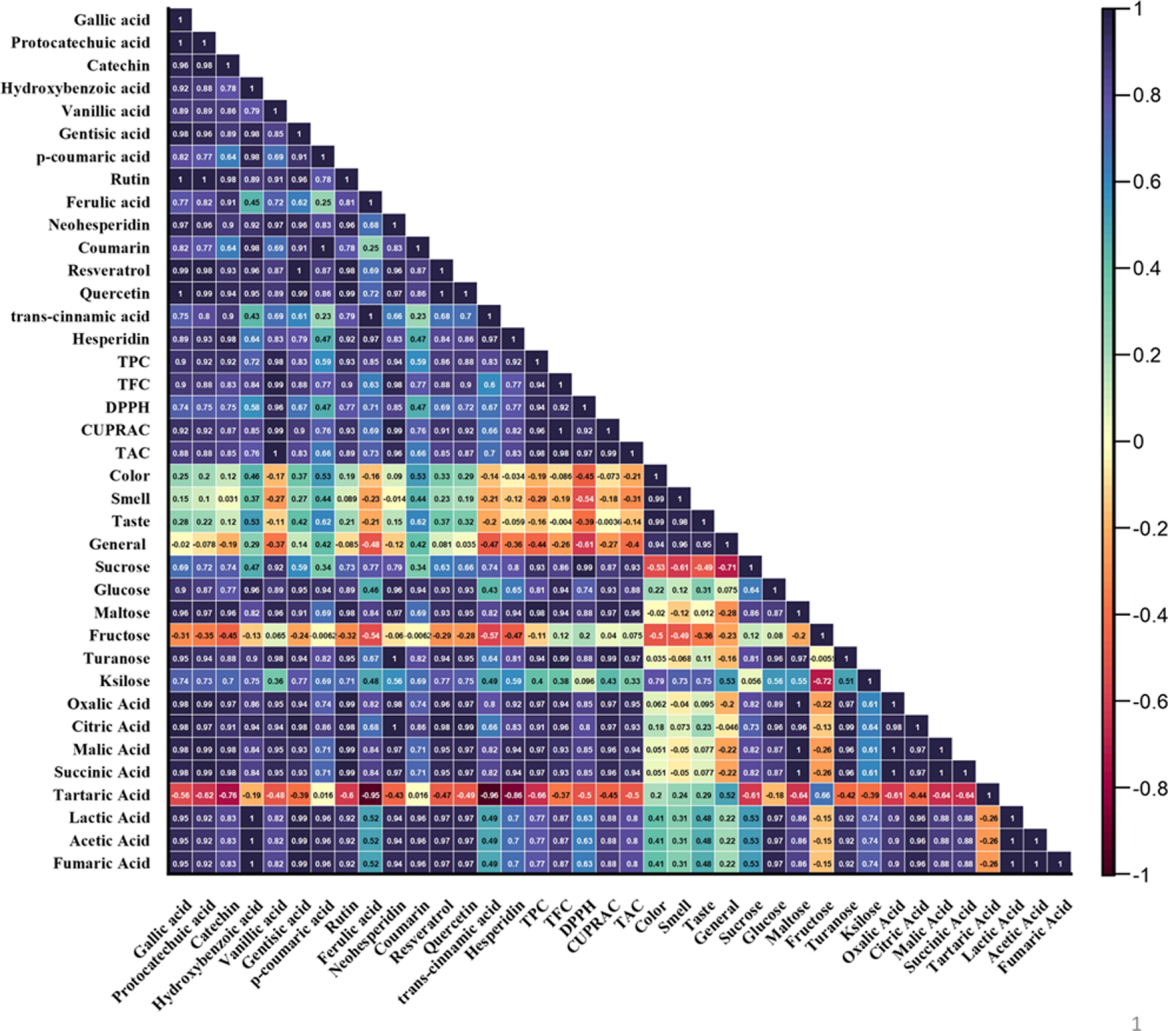
Pearson correlation coefficients for bioactive compounds, organic acids, sugars, and phenolic compounds and sensory analysis.

Anthocyanins are water-soluble colored pigments and form purple, red, and blue colors in fruits and vegetables.^47^ The TAC of bee-butter-fortified functional poppy sherbet samples on days 0, 7, 14, and 21 is given in Figure 5. Total anthocyanin contents in the samples during the storage period were 40.73 ± 0.70 mg Cy-3-gly/mL, 40.03 ± 0.69 mg Cy-3-gly/mL, 37.06 ± 1.11 mg Cy-3-gly/mL, and 35.77 ± 0, respectively. However, it was found that the TAC decreased when it was stored for 21 days. A high positive correlation was found between TAC with vanillic acid (*r* = 1), neohesperidin (*r* = 0.96), and TPC-TFC (*r* = 0.98) (Figure 6). In a study, TAC was found to be 774.49 mg cyn-3-glu/kg in vacuum-processed while TAC was found to be 571.31 mg cyn-3-glu/kg in the traditional method poppy sherbet, and researchers indicated that poppy sherbet is an important source of anthocyanins.^39^ In another study, the total monomer anthocyanin content was found to be 127.23 ± 1.57 mg Cy-3-gly/100 mL, 116.87 ± 2.20 mg Cy-3-gly/100 mL, 98.22 ± 1.14 mg Cy-3-gly/100 mL, and 92.45 ± 1.08 mg Cy-3-gly/100 mL, respectively, of poppy sherbet samples during the 30 days of storage. As in our study, a decrease in TAC was also observed during the storage.

### 3.5 Analysis of Phenolic Compounds during Storage

Phenolic compounds have antioxidant, antimicrobial, and anti-inflammatory effects. It has also been reported that it is used in the treatment of diseases such as obesity, cancer, and diabetes.^36,48,49^ It has been reported that poppy and bee bread are rich in phenolic components and bee bread is a functional food.^3,,51^

The polyphenol results of the sherbet samples prepared with poppy and bee bread in the present study are shown in Table 3. Optimized bee bread-fortified functional poppy sherbet samples were analyzed for 17 phenolic compounds on days 0, 7, 14, and 21. Gallic acid was found to be the most abundant of these compounds. In this study, gallic acid, which had the highest amount, showed a significant decrease of (29.21 ± 0.41 to 21.12 ± 0.30 μg/mL) *p* < 0.05 during the storage period. Similarly to this study, in a study where poppy sherbet was evaluated during storage (30 days), as the time increased, protocatechuic acid (1.146 ± 0.048, 1.047 ± 0.038 μg/mL) and gallic acid (23.886 ± 0.164, 23.403 ± 0.343 μg/mL) nonsignificant decreased.^23^ The values of the phenolic compounds gallic acid, gentisic acid, vanillic acid, catechin, ferulic acid, neohesperidin, quercetin, coumarin, *p*-coumaric acid, and hydroxybenzoic acid at the beginning of storage were higher than the values obtained by Aydoğdu et al.^23^ The reason for this difference is thought to be due to the phenolic components of bee bread added to poppy sherbet. Dranca et al. reported that protocatechuic acid, *p*-hydroxybenzoic acid, gallic acid, chlorogenic acid, and vanillic acid were not detected in bee bread, while the highest concentration was kaempferol (31.25 mg/L) followed by myricetin, luteolin, and rosmarinic acid.^50^ Sawicki et al. reported that gallic acid, chlorogenic acid, protocatechuic acid, and routine were detected in bee bread.^52^ Among the phenolic compounds, routine, naringin, resveratrol, and trans-cinnamic acid are seen to be higher in poppy sherbet.^23^ This result may be because these phenolic compounds are higher in poppy. In the present study, naringin and *o*-coumaric acid were not detected in the analyses performed during storage. While coumarin and p-coumaric acid could not be detected after the seventh day; catechin, hydroxybenzoic acid, resveratrol, and trans-cinnamic acid could not be detected on day 21. The increase in ferulic acid on the 7th and 14th days was not statistically significant.

**Table 3:** Change in the Phenolic Content of the UT-PS Sample during Storage^a^

	storage days			
phenolic compounds (μg/mL)	0	7	14	21
gallic acid	29.21 ± 0.41^a^	25.34 ± 0.35^b^	24.97 ± 0.52^b^	21.12 ± 0.30^c^
protocatechuic acid	1.03 ± 0.01^a^	0.85 ± 0.02^b^	0.83 ± 0.02^b^	0.59 ± 0.01^c^
catechin	0.64 ± 0.01^a^	0.45 ± 0.01^b^	0.44 ± 0.01^b^	0.00 ± 0.00
hydroxybenzoic acid	0.15 ± 0.00^a^	0.03 ± 0.00^b^	0.03 ± 0.00^b^	0.00 ± 0.00
vanillic acid	0.03 ± 0.00^a^	0.03 ± 0.00^a^	0.02 ± 0.00^a^	0.02 ± 0.00^a^
gentisic acid	1.36 ± 0.02^a^	0.88 ± 0.01^b^	0.87 ± 0.00^b^	0.60 ± 0.02^c^
*p*-coumaric acid	0.03 ± 0.00	0.00 ± 0.00	0.00 ± 0.00	0.00 ± 0.00
rutin	0.52 ± 0.01^a^	0.48 ± 0.01^b^	0.47 ± 0.01^b^	0.42 ± 0.01^c^
ferulic acid	0.85 ± 0.01^b^	0.9 ± 0.01^a^	0.87 ± 0.00^ab^	0.45 ± 0.01^c^
naringin	n.d	n.d	n.d	n.d
*o*-coumaric acid	n.d	n.d	n.d	n.d
neohesperidin	4.33 ± 0.06^a^	2.91 ± 0.10^b^	1.90 ± 0.07^c^	0.96 ± 0.03^d^
coumarin	0.09 ± 0.00	0.00 ± 0.00	0.00 ± 0.00	0.00 ± 0.00
resveratrol	0.01 ± 0.00^a^	0.01 ± 0.00^b^	0.01 ± 0.00^b^	0.00 ± 0.00
quercetin	0.30 ± 0.01^a^	0.24 ± 0.01^b^	0.24 ± 0.01^b^	0.19 ± 0.01^c^
*trans*-cinnamic acid	0.20 ± 0.01^a^	0.22 ± 0.01^a^	0.22 ± 0.01^a^	0.00 ± 0.00^b^
hesperidin	0.41 ± 0.01^a^	0.40 ± 0.01^a^	0.39 ± 0.01^a^	0.31 ± 0.01^b^
total	39.16 ± 0.57^a^	32.8 ± 0.55^b^	31.33 ± 0.67^b^	24.66 ± 0.38^c^

a The results are presented as mean ± standard deviation. Different letters indicate significant differences among the values within the same row (*p* < 0.05). n.d: not detected.

The TPC in bee bread-poppy sherbet was determined as 39.16 ± 0.57 at the beginning and 24.66 ± 0.38 on the 21st day. It was stated that the TPC of poppy sherbet was 31.388 ± 0.413 at the beginning and 27.152 ± 0.016 on the 20th day,^23^ while the TPC of bee bread was between 5.6 ± 0.1 and 11.4 ± 0.4.^51^ It is stated that dried poppy flower contains 48.09 ± 0.31 mg GAE/g.^3^ In parallel with our study, Aydoğdu et al. detected a significant decrease in the concentration of the total phenolic components during the storage period. In general, it is thought that the decrease in phenolic content may be related to changes in the polymerization of phenolic compounds.^53^

When the linear relationship between the variables was examined, TPC was significantly associated with quercetin (*r* = 1), vanillic acid (*r* = 0.98), ferulic acid (*r* = 0.94), and *p*-coumaric acid (*r* = 0.93). In addition, a positive correlation was observed between TFC and vanillic acid (*r* = 0.98) and neohesperidin (*r* = 0.98) (Figure 6).

### 3.6 Sensory Properties during Storage

**Figure 7:**
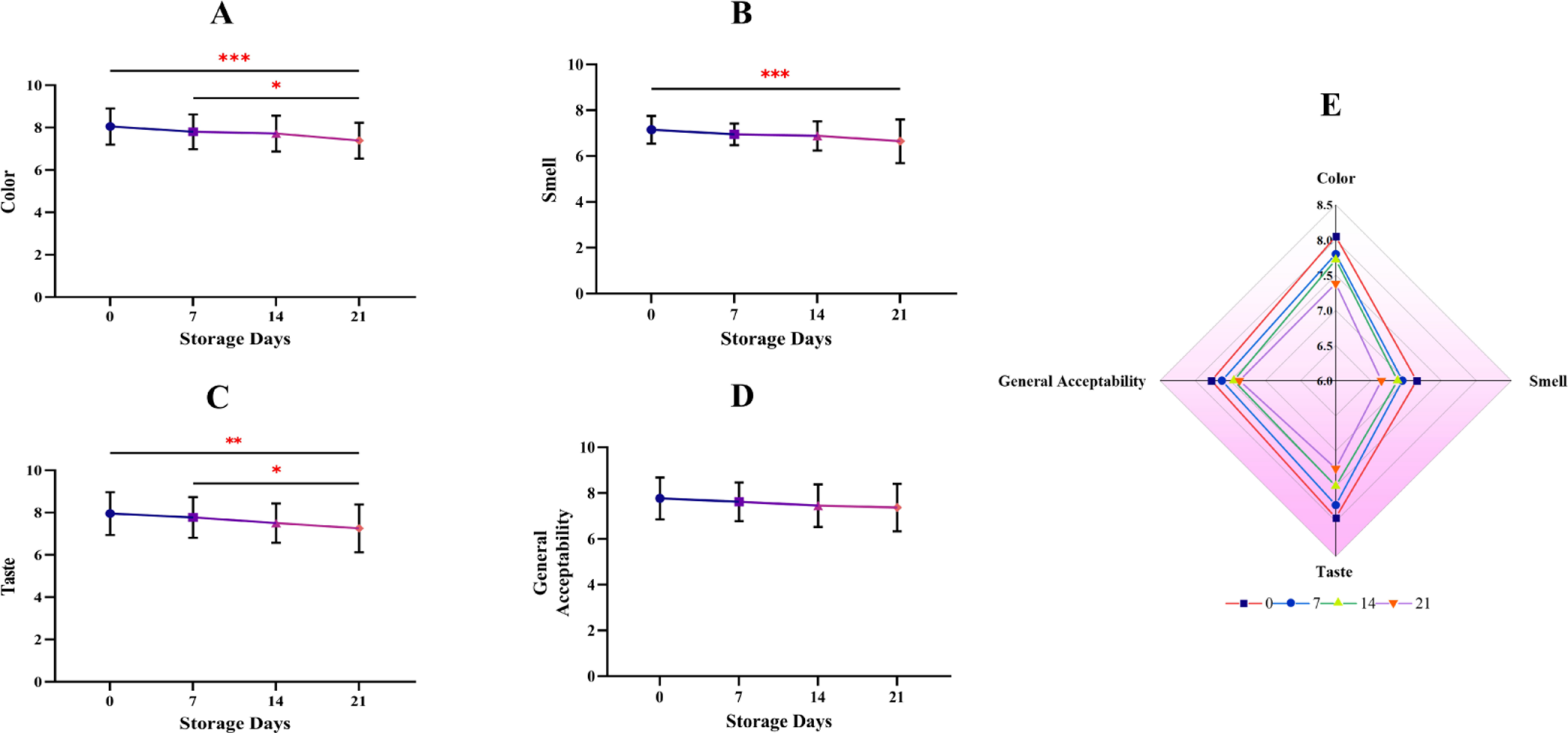
Changes in the sensory properties of the samples during their shelf life.

Sensory analysis results (odor, color, taste, and general acceptability) of sherbet samples evaluated on the 0th, 7th, 14th, and 21st days are given in Figure 7. Color is the most important parameter in consumer taste perception.^54^ When the optimized bee bread-fortified functional poppy sherbet sample was evaluated in terms of color and taste, it showed statistically significant variability after the seventh day (*p* < 0.05). Similarly, in the present study, color losses occurred as a result of the 30-day storage of the poppy sherbet produced by Aydoğdu et al., who reported that color loss was due to the Maillard reaction and losses in anthocyanins.^23^ It was determined that the panelists gave the highest scores to the sherbet samples at the beginning of storage: color (8.05), smell (7.15), taste (7.95), and overall preference (7.77). Similar results were obtained by Aljaloud et al., who investigated the color, consistency, taste, odor, and overall preference properties of raspberry, blueberry, strawberry, and passion fruit-mango sherbets during a 6-month storage period, and they found that the products showed the best sensory properties was at the beginning storage.^55^ In this study, it was observed that there was a tendency for color, odor, taste, and general likability values to decrease with increasing storage time. However, this decrease did not show a statistical difference, and the sherbet samples were still within the general acceptability limits on the 21st day. As seen in Figure 5, there was no strong positive correlation between color, odor, taste, odor, general acceptability values, and all other parameters.

### 3.7 Organic Acid and Sugars

**Figure 8:**
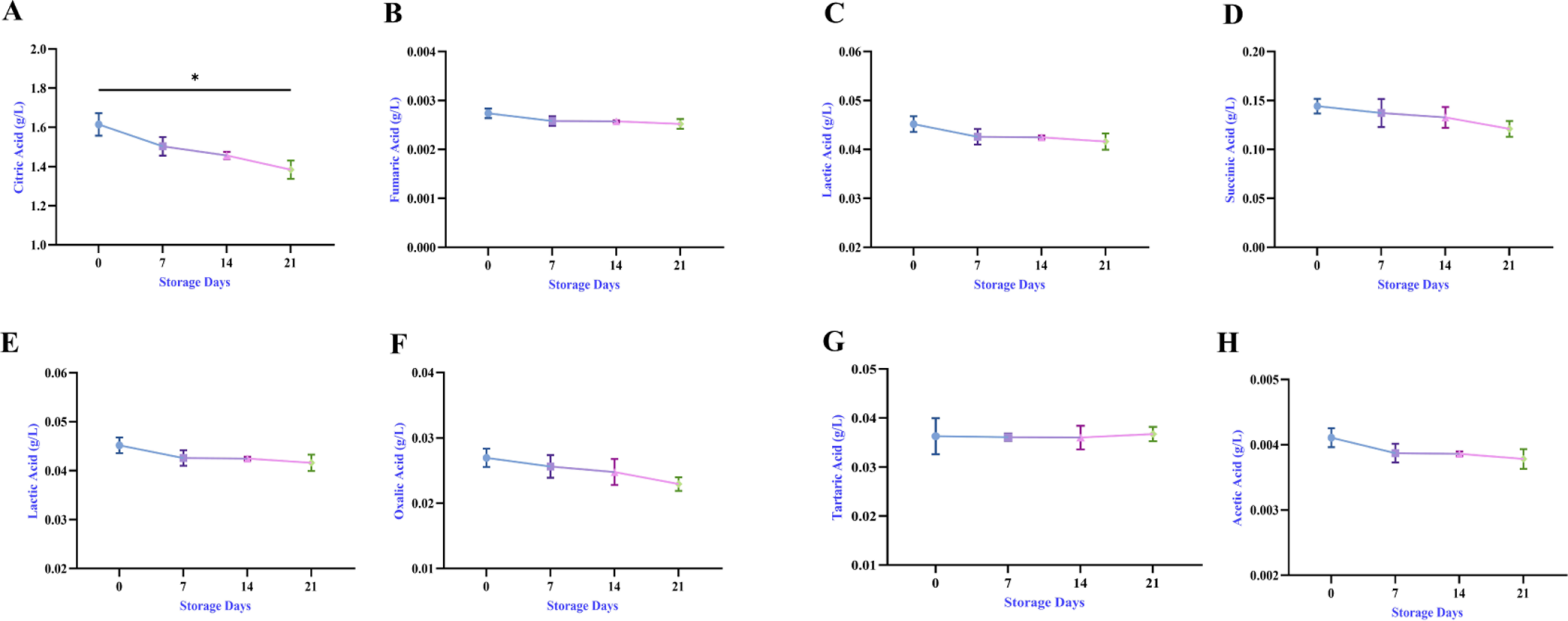
Storage of organic acid compounds (A–H).

**Figure 9:**
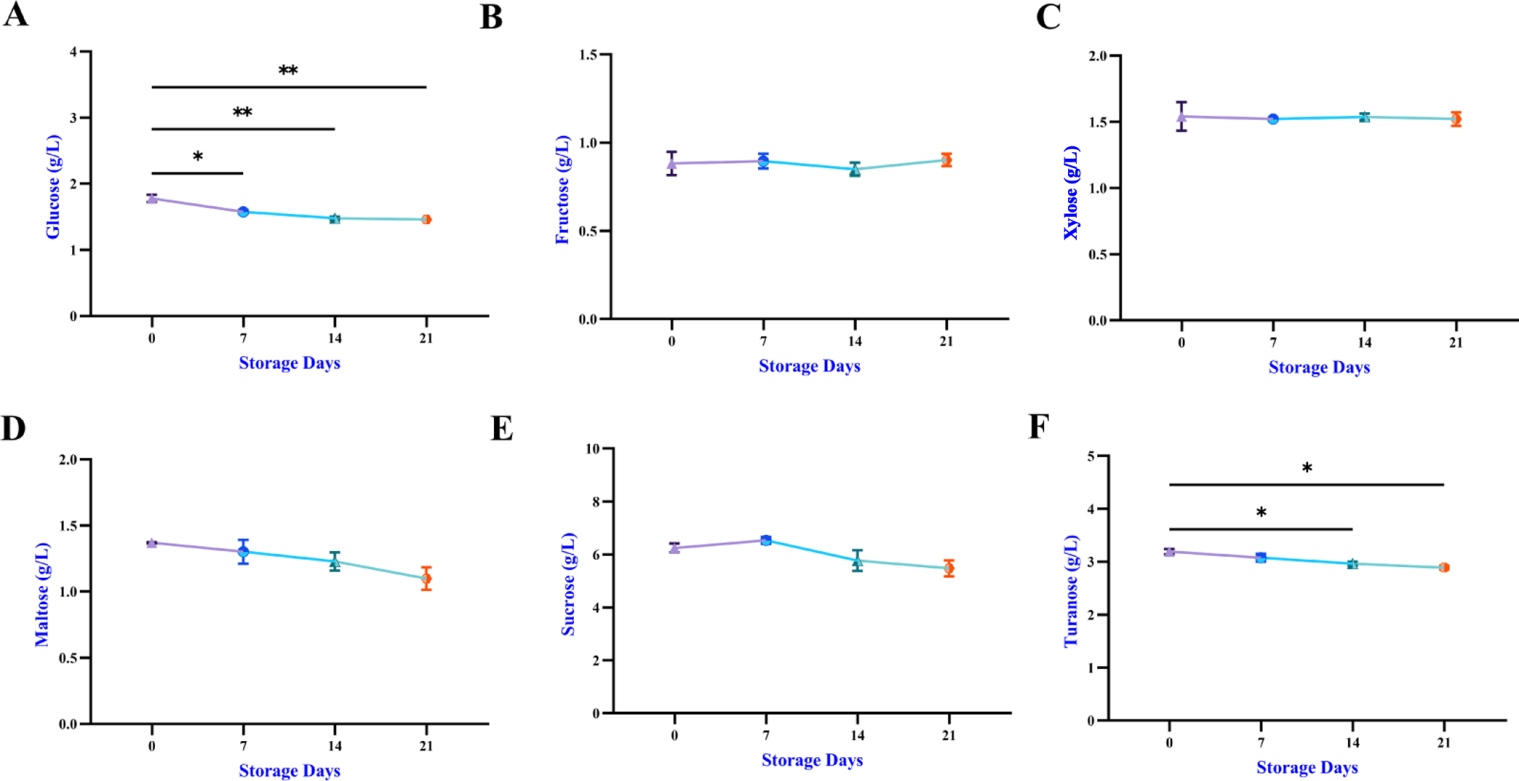
Storage sugar components (A–F).

Changes in the amounts of organic acids and sugar during storage are shown in Figure 8 and Figure 9. While citric acid (1.615 g/L) was found in the highest amount of organic acid, sucrose (6.246 g/L) was found in the highest amount of sugar components on the zeroth day. All sugars detected decreased during storage, except fructose, sucrose, and xylose. The amount of fructose increased by 0.013 g/L on the 7th day, decreased by 0.046 g/L on the 14th day, and increased by 0.20 g/L from the beginning on the 21st day. Decreasing glucose and turanose amounts were found to be statistically significant.

In the present research, bee bread was added to poppy sherbet, and the product also contained sugar and organic acids originating from bee bread. Dranca et al. found fructose (19.73%), glucose (8.82%), melezitose (0.97%), raffinose (0.96%), acetic acid (10.4 g/kg), gluconic acid (79.2 g/kg), propionic acid (1.30 g/kg), butyric acid (0.33 g/kg), and formic acid (6.75 g/kg) in bee bread.^50^ Significant correlations were observed in TPC between sucrose (*r* = 0.93), maltose (*r* = 0.98), and turanose (*r* = 0.94). A positive correlation was observed between TFC and turanose (*r* = 0.99), maltose (*r* = 0.94), and glucose (*r* = 0.94; Figure 6).

Most organic acids showed a decreasing trend, depending on the storage process. A positive correlation was found between TPC and oxalic, malic, and succinic acids (*r* = 0.97). TFC was extremely positively correlated with citric (*r* = 0.96), oxalic (*r* = 0.94), malic, and succinic acids (*r* = 0.93). Other significant correlations were observed between lactic, acetic, and fumaric acids with hydroxybenzoic acid (*r* = 1).

The results obtained are appropriate for the findings in the literature. Ergün et al. reported that the sugar amounts of blueberry and mulberry sherbets decreased significantly after 60 days of storage.^56^ Evrendilek et al. did not determine a significant change in organic acid values (acetic, formic, and fumaric acid) as a result of the 25-day storage of licorice beverages with hydrostatic pressure.^57^

### 3.8 Analysis of Antimicrobial Activity

MIC values of bee-butter-fortified functional poppy sherbet against Gram-negative and Gram-positive bacteria after 21 days of storage were investigated by broth microdilution test (Table 4). While the MIC values of *B. cereus*, *E. faecalis*, and *E. coli* were found to be the same in sherbet samples stored for 0, 7, 14, and 21 days, MIC values against *M. luteus* were determined to be lower. The inhibitory effects of the sherbet against *S. aureus*, *M. luteus*, *P. aeruginosa*, and *K. pneumoniae* were higher at the beginning of storage. In addition, the fact that MBK results were close to/the same as the MIC results showed that this substance has a bactericidal effect on all of the bacteria studied. The antibacterial effects of the samples stored for 0, 7, 14, and 21 days at concentrations ranging from 100% to 6.25% were determined by the disk diffusion method. At the end of the study, zone diameter was determined only in *M. luteus*.

**Table 4:** MIC and MBC Results for 0, 7, 14, and 21 Days

	0 (%)		7 (%)		14 (%)		21 (%)	
bacteria	MIC	MBC	MIC	MBC	MIC	MBC	MIC	MBC
*B. cereus*	50	50	50	50	50	50	50	50
*S. aureus*	25	50	25	50	25	50	25	50
*E. faecalis*	50	50	50	100	50	100	50	100
*M. luteus*	12.5	12.5	25	25	25	25	25	25
*P. vulgaris*	25	50	50	50	50	25	50	50
*P. aeruginosa*	25	50	50	50	50	50	50	50
*E. coli*	50	100	50	100	50	100	50	100
*K. pneumoniae*	25	25	50	50	50	25	50	25

**Figure 10:**
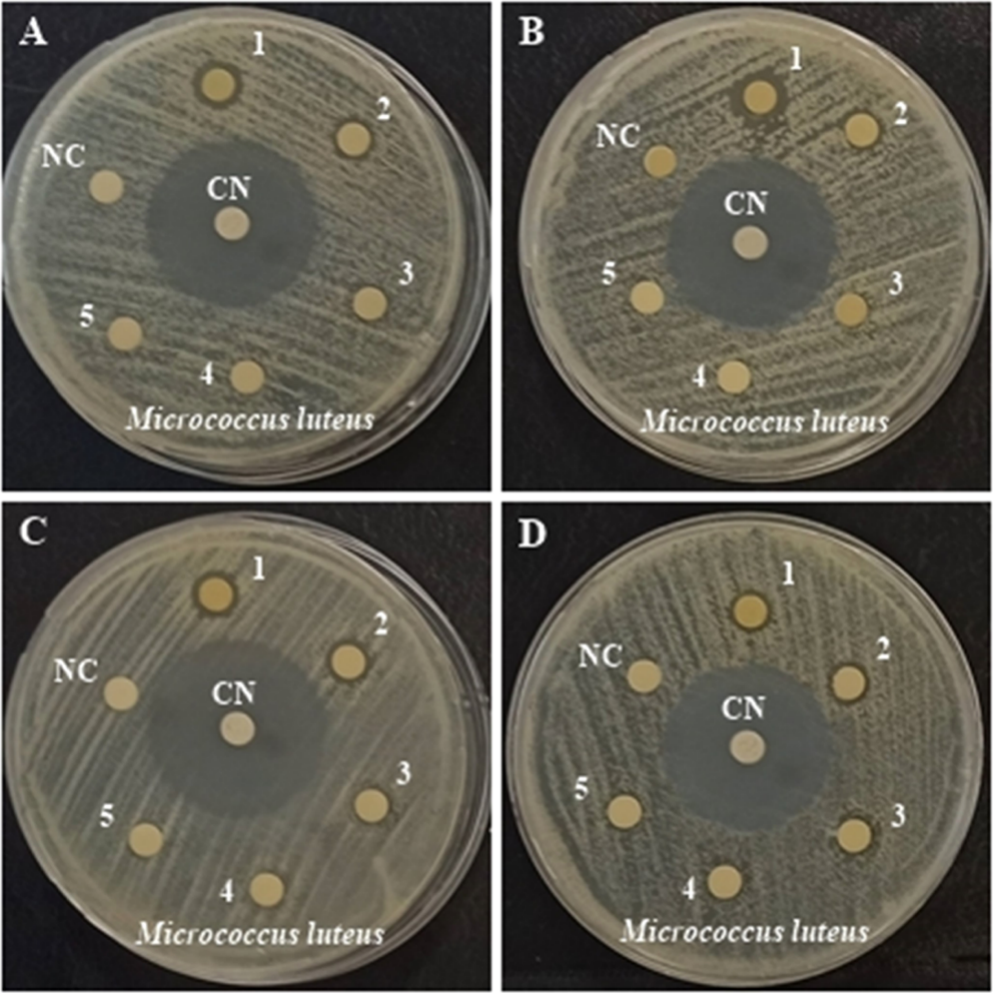
Antibacterial activity of five different concentrations (1:100, 2:50, 3:25, 4:12.5, 5:6.25%) of 0th- (A), 7th- (B), 14th- (C) and 21st-day (D) samples against *M. luteus*. This figure was photographed by Berna Erdal.

It is important that the zone diameter was detected at 25% concentration of 14- and 21-day storage samples. Considering that the lowest MIC and MBC values are seen in *M. luteus*, it can be said that bee bread-fortified functional poppy sherbet samples show an effective antibacterial activity, especially on this bacterium (Figure 10). The lowest MIC and MBK values were detected in *M. luteus*, and it was determined that all samples stored for 0, 7, 14, and 21 days showed important antibacterial activity. When *M. luteus* MIC/MBC results of the 0-, 7-, 14-, and 21st-day samples were compared, it was observed that the antibacterial effect of *M. luteus* decreased depending on the increase in storage time (Table 5). Similarly, in the present study, Marsoul et al. reported that *P. rhoeasL*. flower extracts (maceration) against *E. coli* have higher MIC values than *K. pneumoniae*. Poppy is thought to have antimicrobial activity because it contains phenolic, flavonoids, lignans, fatty acids, anthocyanins, and isoquinoline alkaloids.^4^ In a similar study, *Micrococcus roseus* was given 8.1 ± 3.4 mm zone diameter antibacterial activities of aqueous decoctions of poppy.^58^ It was found that antimicrobial activity decreased as the storage time increased.

**Table 5:** Inhibition Zone Diameters for *M. luteus* in mm^a^

storage days	concentration (%)	zone diameter (mean ± SD, mm)
0	100	10.08 ± 0.28
	50	8.150 ± 0.15
	25	ND
7	100	11.08 ± 0.28
	50	8.03 ± 0.09
	25	ND
14	100	9.13 ± 0.44
	50	8.10 ± 0.60
	25	7.03 ± 0.10
21	100	9.20 ± 0.27
	50	7.98 ± 0.13
	25	7.01 ± 0.09

a SD: Standard deviation, ND: None diameter.

## 4 Conclusions

In the present paper, the effects of ultrasound on the antioxidant activity of capacity (DPPH and CUPRAC), antimicrobial activity, phenolic compounds, organic acid and sugar composition, and sensory properties optimizing bee bread-fortified functional poppy sherbet were evaluated. It was determined that there was a decrease in the total monomeric anthocyanin content during the 21-day storage period. Seventeen phenolic compounds were detected in optimized bee bread-fortified functional poppy sherbet. Gallic acid, which had the highest amount, showed a significant decrease after 21 days. All sugars detected decreased during storage, except fructose, sucrose, and xylose. The lowest MIC and MBK values were detected in *M. luteus*. It was determined that the score the panelists gave to the bee bread-fortified functional poppy sherbet decreased during storage. Based on these results, further research (toxicity, anticarcinogenicity) is needed to better elucidate the impact of the ultrasonic process and the storage of the sherbet. The data we have obtained for poppy sherbet enriched with bee bread as a functional product will lead to in vivo studies.

## Data Availability

The data supporting this study’s findings are available upon request from the corresponding author. The data are not publicly available due to privacy or ethical restrictions.
